# Overnutrition in Indian Children: Challenges and Opportunities

**DOI:** 10.3389/fpubh.2022.814900

**Published:** 2022-03-03

**Authors:** Prema Ramachandran, K. Kalaivani

**Affiliations:** Nutrition Foundation of India, New Delhi, India

**Keywords:** Indian children, pre-school children, school age children, over-nutrition, BMI-for-age, WHO growth standards

## Abstract

Global and Indian data indicate that children from all the segments of population face dual nutrition burden and related health consequences. Long-term cohort studies have shown that both the under- and overnutrition are risk factors for overnutrition and non-communicable diseases in adult life. Halting the rise in overnutrition is one of the Sustainable Development Goal (SDG) targets to be achieved by 2030. With the development and inclusion of body mass index (BMI)-for-age in the WHO child growth standards, it has become possible to assess over- and undernutrition in short-statured children. In India, the Annual Health Survey (AHS) (2014) and the District Level Household Survey 4 (DLHS4) (2013) undertook measurement of height/length and weight (AHS 557016 and DLHS4 295663) in the 0–18-year of school-age children from selected households. Prevalence of overnutrition in 0–18-year children was calculated from these two surveys by using the WHO standards for BMI-for-age (BMI-for-age z scores (BAZ) > +2 in 0–5 and BAZ > +1 in 5–18-year children) as well as uniform norms of either > +1 or > +2 BAZ across 0–18-year children. An attempt was made to explore the policy and program implications of using different norms for assessing overnutrition in preschool and school age children in the Indian context. Body mass index-for-age curve for the 0–18-year Indian children was calculated and compared with the WHO BMI-for-age curve. Across 0–18-year children, the mean BMI-for-age of Indian boys and girls was lower than the mean of the WHO standards, but the trajectory followed was similar. Therefore, Indian high-risk under- and overnourished children can be monitored by using the WHO BMI-for-age curve. Irrespective of the cutoff used for BMI-for-age, prevalence of overnutrition was higher in preschool as compared to school-age children. Overnourished school-age children outnumbered preschool children, especially if the WHO cutoffs were used. The school health system may find it difficult to implement programs that aimed at detection and management of large number of overnourished children. If uniform norm of BAZ > +1 was used, prevalence of overnutrition in preschool children was high and almost similar to undernutrition. Currently, nutrition programs for preschool children are focused on undernutrition and they may find it difficult to manage program focused on overnutrition in large number of children. If the uniform norm of BAZ > +2 was used, both the prevalence of overnutrition and number of children requiring intervention were relatively low in all the age groups. The existing preschool and school nutrition programs can take up an integrated program aimed at early detection and effective management of both the under- (BAZ < −2) and overnutrition (BAZ > +2) in 0–18-year children and strive to achieve the SDG targets.

## Introduction

Six decades ago, the majority of Indians were poor and food insecure; over two-thirds of children and adults were undernourished. The country initiated multipronged interventions to reduce poverty, improve household food security, and provide food supplements to vulnerable groups such as pregnant women and children. India has been undergoing socioeconomic, demographic, nutrition, and health transition. In the last two decades, there has been relatively rapid gross domestic product (GDP) growth, rise in per capita income, and reduction in poverty ratio (1, 2). The last four decades had witnessed a substantial reduction in undernutrition rates across all the age groups; however, stunting, underweight, and wasting rates in Indian children are still among the highest in the world (3–15). In addition, the country witnessed an increase in the overnutrition rates in children (10–12, 14, 15). With the emergence of the dual nutrition burden in India, there was growing concern among public health nutritionists and pediatricians that if the weight for age is used as the indicator for assessing nutritional status, the short-statured Indian children who were overweight for their age and height will not be recognized as overnourished and opportunity to initiate early interventions will be missed.

The World Health Organization Multi country Growth Reference Standards (WHO MGRS) (16) and the WHO child growth standards (17) published in 2007 incorporated a new indicator—body mass index (BMI)-for-age—which takes both the current age and stature into account for calculating nutritional status in children. The WHO standards define undernutrition in 0–18-year children as Body Mass Index for age z scores (BAZ) < −2, but overnutrition in 0–5-year children is defined as BAZ > +2 and in 5–19-year children is defined as BAZ > +1 (16, 17). The WHO has set the nutrition targets for both the under- and overnutrition in children as assessed by BMI-for-age to be achieved by 2025 (18); all the countries are signatories to the Sustainable Development Goal (SDG) nutrition targets to be achieved by 2030 (19).

Given the impetus of monitoring the WHO/SDG targets, global and national agencies have been collecting, collating, and reporting data on dual nutrition burden in under-five children by using the WHO MGRS standards (20). Globally, prevalence of wasting is 6.7% and overweight is 5.4%. Wasting rates are lowest in North America (0.1%) and highest in Asia 8.9%; in contrast, overnutrition rates are highest in North America (7.3%) and lowest in Asia (4.5%) (20). Prevalence of wasting and overnutrition in 5–18-year children had also been reported by various agencies, but a comparison of data between countries or periods is difficult because of the use of different criteria (weight for height or BMI-for-age) and different cutoff used for overnutrition (> +2 SD in 0–5 years, > +2 SD in 0–18 years, and > +1 SD in 5–18 years) (20, 21). There have not been any studies to assess the potential impact of the difference in the WHO criteria for overnutrition between preschool and school-age children, on the prevalence of overnutrition, and the number of children requiring intervention for overnutrition.

In India, undernutrition rates in children are high; two of the largest food supplementation programs in the world [the Integrated Child Development Services (ICDSs) for preschool children and the Mid-Day Meal (MDM) for school children] aimed at bridging the dietary energy gap in children are being implemented in India. To achieve the SDG targets, India has to undertake programs focusing on prevention, detection, and management of both the under- and overnutrition in children. There are ample data on the prevalence of undernutrition in children, but there is a paucity of data on the prevalence of overnutrition in children. It is essential to have data on the prevalence and number of preschool and school-age children who would need interventions to halt and reverse the rise in overnutrition. In 2013–2014, India carried out two major surveys to assess the nutritional status of 0–18-year children. Data from these surveys were analyzed to calculate the prevalence of overnutrition in children by using the WHO BMI-for-age standards. The results of the data analysis and the potential policy and program implications of using the different BAZ cutoffs for overnutrition in preschool and school-age children in India are discussed in the manuscript.

## Materials and Methods

The Annual Health Survey (AHS) Clinical, Anthropometric and Biochemical Component (CAB) (2014) (14) and the District Level Household Survey 4 (DLHS 4) (2013) (12) undertook assessment of nutritional status of over 900,000 children in the 0–18-year age group. The AHS CAB (14) and DLHS 4 (12) undertook height and weight measurements in all the 0–18-year children from households selected for the survey. The sampling frame for the surveys had been prepared by the International Institute for Population Sciences (IIPS), Mumbai and Office of the Registrar General of Registrar General of India (RGI), New Delhi. The survey personnel was paraprofessionals and was trained in administering the survey instruments and taking measurements. All the anthropometric equipment for the survey were centrally procured and tested for accuracy before being sent to the survey agencies. Prior to initiation of the survey, all the paraprofessionals recruited by various agencies undertaking the survey in different states were trained in undertaking measurement of length/height and weight. The length/height measurements in the survey were taken by personnel with required accuracy in measurements. As an internal quality assurance procedure, duplicate measurements were carried out in randomly selected 10% of the children measured.

Data from the AHS CAB were obtained from the Ministry of Health and Family Welfare and data from the DLHS4 were obtained from the IIPS. Data were cleaned and analyzed by using the WHO Anthro and the WHO AnthroPlus software and the SPSS software version 26 (SPSS Incorporation, Chicago, Illinois, USA).

The BMI-for-age curve for the 0–18-year Indian children was calculated for mean, 2.3 centile, and 97.7 centile; these were compared with the WHO BMI-for-age curve. Prevalence of overnutrition in 0–18-year children was calculated using the WHO standards (overnutrition in 0–4-year children BAZ > +2 and in 5–19-year children BAZ > +1). In addition, overnutrition rates were calculated by using uniform cutoff of BAZ > +1 as well as BAZ +2 in 0–18-year children.

## Results

The number of children in the 0–18-year age group in the selected households in the DLHS 4 and the AHS CAB surveys and the number of children in whom height/length and weight measurements were taken are given in Table 1. Almost all the children in the selected households were measured.

**Table 1:** Total number of children with height and weight measured.

**DLHS 4 and AHS CAB**					
**Age**	**Measured**	**Not present**	**Refused**	**Other reasons**	**Total**
0–4 yrs	205,004	7,633	949	288	213,874
5–9 yrs	224,993	12,646	779	349	238,767
10–14 yrs	228,455	15,877	847	390	245,569
15–18 yrs	194,227	14,108	773	360	209,468
Total	852,679	50,264	3,348	1,387	907,678

**Figure 1:**
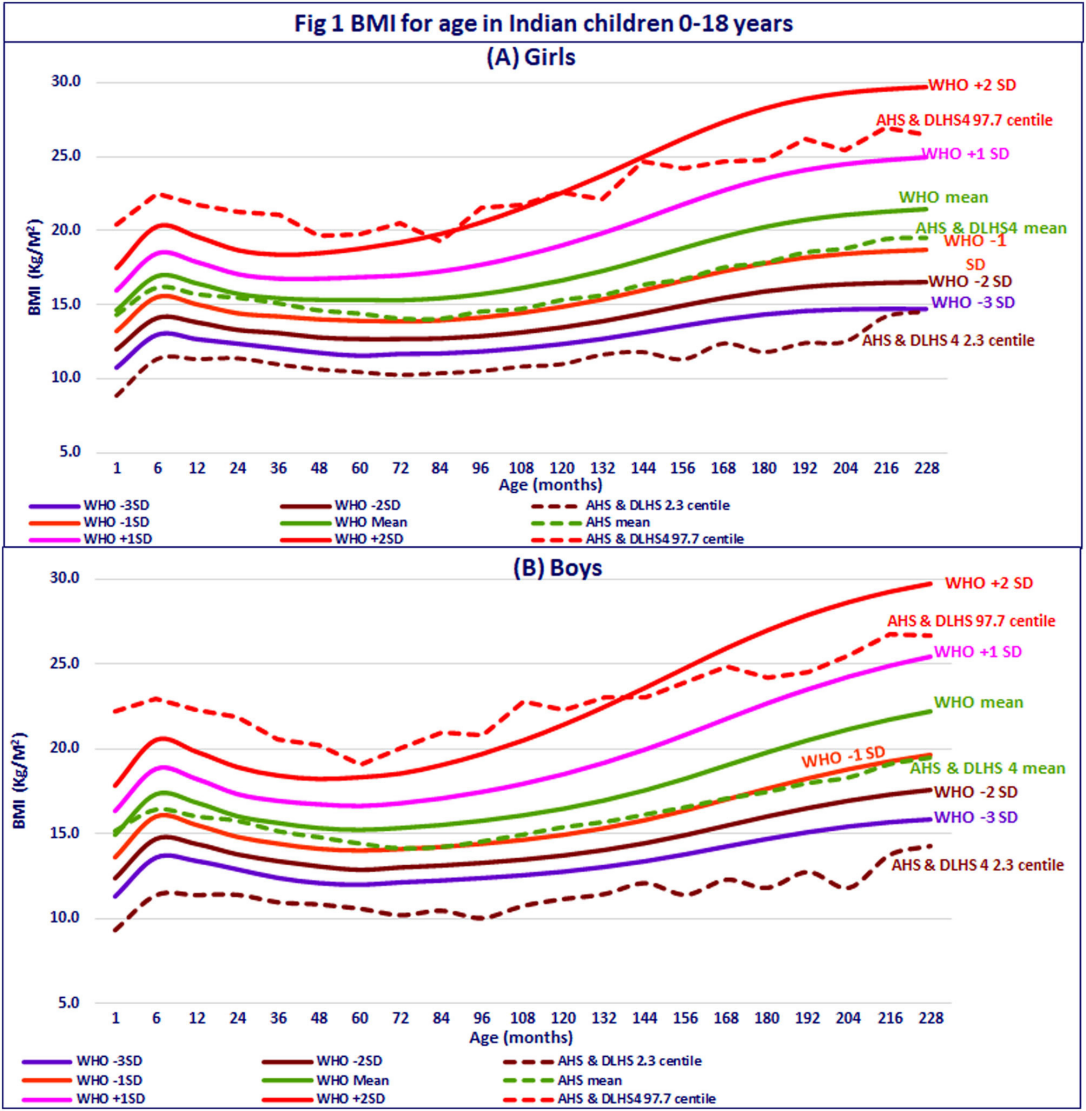
BMI for age in Indian children 0–18 years. **(A)** Girls. **(B)** Boys.

Body mass index-for-age curve for the 0–18-year Indian children was calculated and compared with the WHO BMI-for-age curve. In the first year, the mean BMI-for-age of Indian infants was along the WHO mean for BMI-for-age; thereafter, till 5 years, the mean BMI-for-age curve of Indian children was between the mean and −1 SD of the WHO standards; in 5–18-year Indian children, the mean BMI-for-age of Indian children was along the WHO −1 SD. The 2.3 centile (corresponding to −2 SD) of Indian children is far below the WHO −3 SD till 18 years of age (Figures 1A,B). The 97.7 centile (corresponding to +2 SD) of Indian children was higher than the +2 SD line of the WHO standards in 0–1 year, but declined in 1–9 years; at 18 years, it was at +1 SD of the WHO growth standards. Across 0–18 years, the mean BMI-for-age of Indian boys and girls was lower than the mean of the WHO standards, but the trajectory followed was similar. Therefore, the WHO BMI-for-age chart can be used for growth monitoring using the BMI-for-age in Indian children.

**Figure 2:**
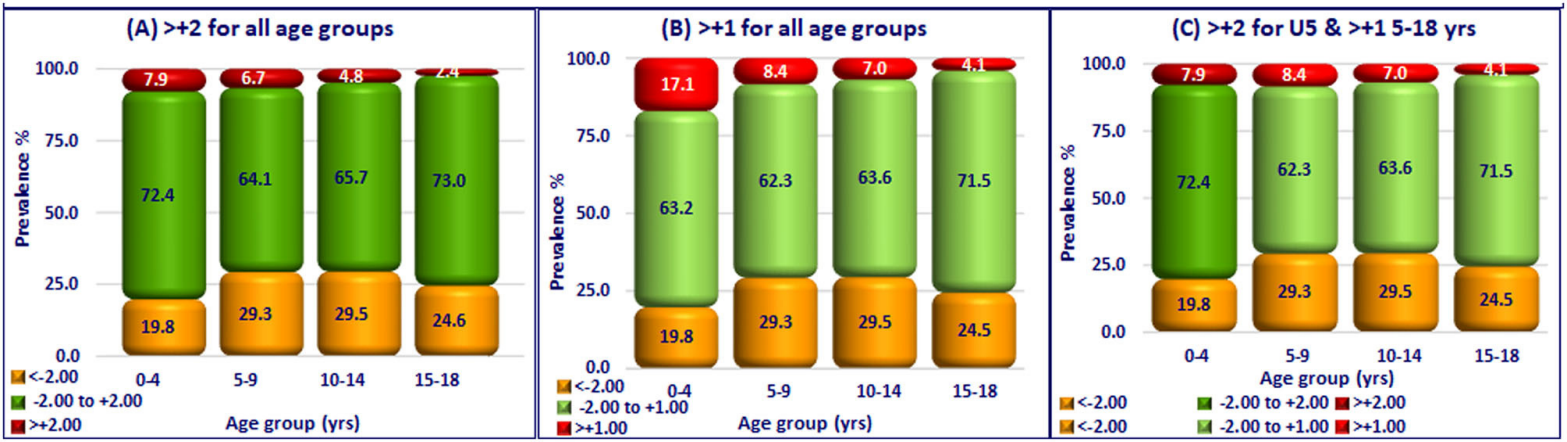
Prevalence of over-nutrition in Indian children (0–18 years). **(A)** > +2 for all age groups. **(B)** > +1 for all age groups. **(C)** > +2 for U5 & > +15–18 yrs.

Prevalance of wasting was higher than the prevalence of overnutrition in all the age groups, indicating that undernutrition continues to be a major public health problem in India. Irrespective of the cutoff used for BMI-for-age, prevalence of overnutrition was higher in preschool as compared to school-age children. If uniform norm of BAZ > +1 was used, prevalence of overnutrition in preschool children was almost similar to undernutrition. If uniform norm of BAZ > +2 was used, both the prevalence of overnutrition and number of children requiring intervention were relatively low in all the age groups (Figures 2A–C).

## Discussion

### Monitoring Progress Toward the SDG Target of Halting the Rise in Overnutrition

Globally, over the last three decades, there has been a reduction in undernutrition and an increase in overnutrition in children. It is estimated that currently, among preschool children, 45 million were wasted and 39 million were overweight; 340 million school-age children were overweight or obese (20). There are variations between countries in the use of standards for defining overnutrition, availability of national survey data on the prevalence of overnutrition, and estimated prevalence of overnutrition. Taking these into account, the SDG sets a feasible target of halting the rise in overnutrition. All the countries can report changes in overnutrition rates in children by using whatever standards they have been using; the WHO/United Nations Development Programme (UNDP) can monitor the progress toward halting overnutrition, despite the variations between different countries. Efforts to set and use uniform standards for defining overnutrition in 0–18-year children and periodic estimation and reporting of the prevalence of overnutrition across countries are being initiated as a part of the SDG monitoring. While the system was getting organized, coronavirus disease 2019 (COVID-19) pandemic occurred; the focus shifted to prevention and management of COVID-19 pandemic, pandemic-related increase in food insecurity, and undernutrition. However, the coming years are likely to see a substantial improvement in reporting both the wasting and overnutrition in children by using BMI-for-age standards. This, in turn, will improve the operationalization of focused programs aimed at halting overnutrition in children.

### Growth Monitoring of High-Risk Children by Using BMI-For-Age

Data from longitudinal studies in India and globally showed that both the under- and overnutrition in children are risk factors for overnutrition and non-communicable diseases in adult life (21). Given this scenario, both the undernourished and overnourished children require BMI monitoring. BMI-for-age charts by the WHO provide a ready graphic tool for detecting deviations from the trajectory of BMI-for-age in individual child over time. Indian children had a lower BMI for any given age as compared to the WHO growth reference standards across all the age groups, but followed a similar smooth trajectory between 0 and 18 years (Figures 1A,B). Plotting BMI for the age of the individual child in the WHO standards is a viable and feasible option for early detection of deviation in BMI for age in children and initiating effective interventions. The continued monitoring of the child by using BMI for age trajectory will also help in assessing the impact of the intervention.

It is well-documented that the Indians, including children, have lower muscle mass as compared to the Caucasians. This might be one of the reasons for the lower BMI for age in Indian children. Whether Indian children also have a lower fat mass as compared to Caucasians is not well-documented. Body composition studies in Indian children are required to document the contribution of muscle mass and fat mass in the lower BMI for age in Indian children. Long-term longitudinal studies are also needed to assess appropriate BMI for age cutoff, which is associated with an increased risk of overnutrition and non-communicable disease in adult life.

### Assessment of Nutritional Status in Dual Nutrition Burden Era

Five decades ago, the focus of intervention programs in India was mainly on prevention, detection, and management of undernutrition in children. Till 2007, the WHO/National Center for Health Statistics (NCHS) growth standards (22) were used in India for early detection and effective management of undernourished children in pediatric and public health nutrition practice. The WHO-NCHS growth standards defined undernutrition as height-for-age or weight-for-age or weight-for-height of < −2 SD; overweight was defined as weight-for-age > +2 SD. Because of the availability of the balances and ease of weighing children, weight-for-age was the most widely used parameter for assessing the nutritional status of children. In hospital settings, weight and height were measured; stunted and underweight children were identified. In settings where accurate age ascertainment was a problem, but height and weight measurement were possible, weight-for-height as an age-independent indicator of nutritional status in children was used for detection of undernutrition. With the emergence of dual nutrition burden, it was recognized that if the WHO/NCHS weight-for-age standards were used, short-statured (e.g., Indian) children who were overweight for their current height will be misclassified as normal or underweight and an opportunity for early detection and effective management of overnutrition will be missed. In the dual nutrition burden era, it was essential to have growth standards that take both the current age and height into account while assessing nutritional status.

The USA was the first to develop the Centers for Disease Control and Prevention (CDC) standards for BMI-for-age for assessment of both the under- and overnutrition in children (23). BMI-for-age growth charts used 85th centile of the NCHS data as the cutoff for overnutrition and 95th centile as the cutoff for obesity. These cutoffs coincided with the accepted adult cutoff of BMI 25 and 30 kg/m^2^ for overnutrition and obesity, respectively (23). In the USA, wasting rates in children were low; the progressive increase in overnutrition rates in children over two decades was recognized as a major health problem to be combatted. Therefore, the CDC standards by using BAZ of +1 for defining overnutrition in children across 0–18 years was accepted and used in the USA.

**Figure 3:**
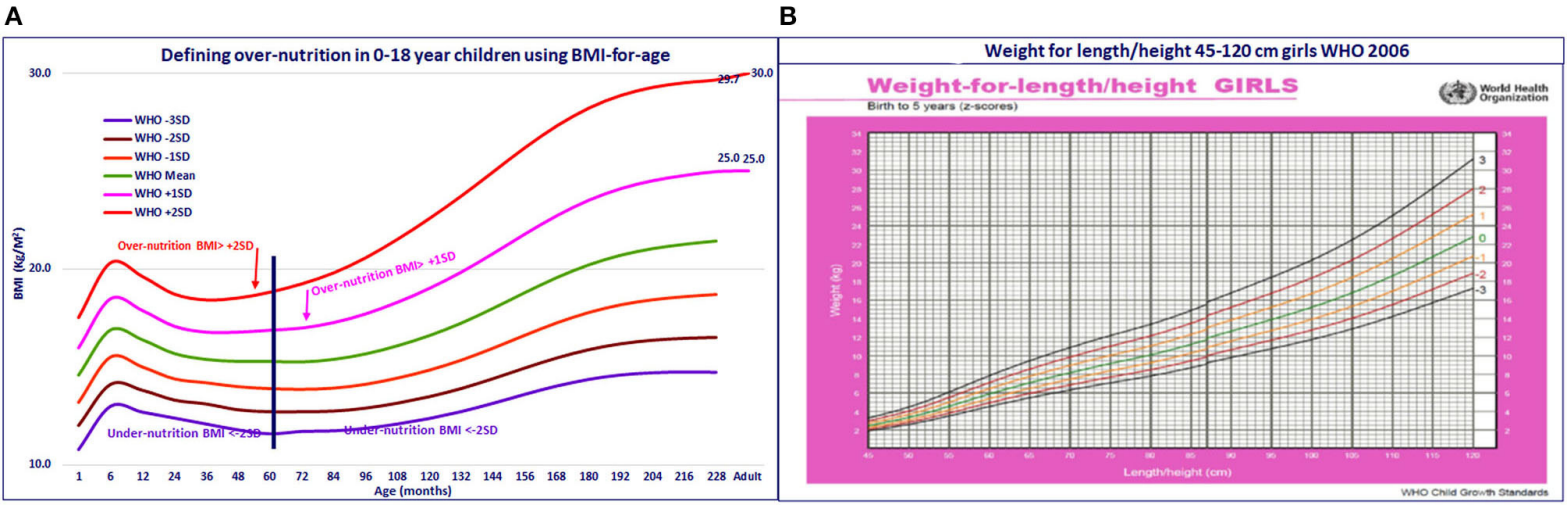
**(A)** Defining over-nutrition in 0–18 year children using BMI-for-age. **(B)** Weight for length/height 45–120 cm girls WHO 2006.

Realizing the urgent need to develop a single global growth reference standard for height-, weight-, and BMI-for-age for preschool and school-age children, the WHO took up the arduous task of reconstructing the 1977 NCHS/WHO growth reference standards (for 0–24 years) by using the original sample (non-obese US children with expected heights), supplemented with data from the WHO MGRS (to facilitate a smooth transition at 5 years), applied state-of-the-art statistical methods, and constructed the new 0–18-year WHO Child Growth Standards (24–26). In the harmonized NCHS/MGRS growth curve, across 0–18 years, undernutrition was defined as z-scores of height-, weight-, or BMI-for-age < −2. However, BAZ scores for overnutrition varied; in 0–5 years, BAZ > +2, as already defined in the WHO MGRS standards, was used (16). For 5–18-year children, BAZ of > +1, which coincided with an accepted adult cutoff of BMI 25 for overnutrition was used (17). As a result, there was an abrupt and substantial difference in the cutoff values for overnutrition for BMI-for-age at 60 months, but the transition from adolescent to adult cutoff BMI for overnutrition was smooth (Figure 3A).

During the development of the WHO growth reference standards, it became obvious that weight-for-height varies with age and is not an age-independent indicator. Further calculation showed that BMI-for-age is a sigma-shaped curve in the 0–18-year age group (Figures 1, 3). Given these findings, the WHO provided the standards for weight-for-age, height-for-age, BMI-for-age, and weight-for-height (27), but recommended the use of BMI-for-age as the preferred indicator for assessment of wasting and overnutrition, especially in short-statured children. However, some of the WHO guidelines continue to indicate both the weight-for-height and BMI-for-age as indicators for assessing wasting and overweight (28) and others recommend weight-for-height as the indicator for assessment of undernutrition (29). In India, the majority of nutritionists and pediatricians have switched over to the use of BMI-for-age for assessment of nutritional status. Among national surveys, some continue to use weight-for-height instead of BMI-for-age for assessment of wasting and overnutrition in children (9, 11, 30), while others have shifted to use of BMI-for-age (12, 14). This accounts for some of the differences in the reported wasting rates between surveys. There are rapid changes in the growth of children (especially breastfed children) in the critical first 2 years of life (Figures 1, 3); BMI-for-age captures these age-related changes and helps in early detection of deviation in growth from BMI trajectory associated with changes in infant and young child feeding practices. Weight-for-height, being age independent, does not provide these insights (Figure 3B). In view of this, we have preferred to use BMI-for-age for assessment of nutritional status in children.

### Detection and Management of Overnutrition in Preschool Children in India

In India, the ongoing ICDS program aims at:

⮚ Prevention of undernutrition (through food supplementation to all the children at 6–72 months of age).
⮚ Weighing at periodic intervals and monitoring growth for early detection of undernutrition.
⮚ Food supplementation to bridge the gap in energy intake in undernourished children.
⮚ Monitoring the response.

Because of the ready availability of balances and ease of weighing children by using digital balances, the program has been using weight-for-age for assessment of undernutrition. Efforts are underway to operationalize growth monitoring by using the WHO weight-for-age growth charts. The ICDS centers are currently being provided with stature meters and the frontline workers are being trained in measuring height. They have mobiles in which they could calculate BMI for each child. The ICDS centers are being provided with the WHO growth charts. By using the WHO BMI-for-age chart, they can assess whether the child is wasted, normal, or overnourished. Prevalence of undernutrition as assessed by BAZ < −2 is substantially lower (about 20%) as compared to the prevalence of undernutrition as assessed by Weight-for-age z scores (WAZ) < −2 (30–40%). Therefore, using BMI-for-age for defining undernutrition, will reduce the workload of the frontline workers providing additional supplements and care to the undernourished children.

The prevalence of overnutrition in preschool children (BAZ > +2) in India is relatively low (2–6%). If the BAZ > +1 cutoff is used, the prevalence of overnutrition in preschool children is over 17%; this is almost as high as the prevalence of wasting. So far, the focus of the ICDS program has been on the management of undernutrition. It might not be appropriate to tell the community that now overnutrition is also a problem of similar magnitude, especially in view of the fact that the long-term risks associated with overnutrition in preschool children have been documented only when cutoff of BAZ of > +2 is used. Unlike the situation in relation to undernutrition, the interventions to halt and reverse overnutrition in preschool children comprise mostly of nutrition education regarding appropriate feeding. Over time, the ICDS and the health system may be able to shift to using BMI-for-age for assessing nutritional status in children and the health and the ICDS workers may be able to deal with early detection and effective management of both the under- and overnutrition in preschool children. Such a shift in the program may pay rich dividends in terms of reducing both the under- and overnutrition and meeting the SDG targets and also a reduction in risk of adult overnutrition and non-communicable diseases.

### Detection and Management of Overnutrition in School-Age Children in India

Indian school children benefit from the MDM program that aimed at preventing undernutrition by bridging the gap between energy requirement and intake and the school health programs aimed at screening children for health problems. There is a nutrition education component advising the children:

⮚ To consume an adequate balanced meal.
⮚ To have an adequate physical activity for optimal nutrition and health.
⮚ Not to habitually consume high-fat, high-energy and high-salt snacks and high-energy beverages.

Neither the MDM program nor the school health program has an inbuilt component of periodic measurement of height and weight, calculating BMI, detection of under- and overnourished children, and providing individual child-focused management of nutrition problems. The MDM and the school health programs may have to be reorganized, so that they can take-up periodic measurements of height, weight, calculate BMI-for-age, and detect and manage under- and overnourished children.

Reported undernutrition rates in school-age children in India is high (between 25 and 30%). The prevalence of overnutrition in school-age children is low (1–5%), if BAZ of > +2 is used, but the prevalence increases to 5–9% if BAZ > +1 is used. Using BAZ of > +1 in 5–18-year children for defining overnutrition, will, therefore, substantially increase the number of school children requiring intervention for overnutrition. Available data suggest that the risk of overnutrition and non-communicable disease risk are higher in preschool children having BAZ > +2. There is a paucity of data on the risk of overnutrition and non-communicable diseases in adult life in school-age children with BAZ > +1. Taking all these factors into consideration, it might be preferable not to use BAZ > +1 for defining overnutrition in school children in India.

Screening and detecting under- and overnutrition and providing appropriate interventions might be easier in 5–18-year children in school settings. School children may themselves play an important role in height and weight measurement, calculating BMI, and identifying wasted and overnourished children and benefit by learning valuable lessons from the exercise. In school settings, it is possible to provide:

⮚ A second helping of the MDM and treat infections, if present in wasted children.
⮚ To counsel overnourished children to increase physical activity and not to consume energy-dense snacks and beverages frequently.

Though overnutrition rates are lowest in the 15–18 age groups, the figure-conscious adolescents, especially girls, may readily accept and practice interventions to reduce overnutrition.

The existing school health and the MDM programs can provide nutrition and health education regarding optimal diet, physical activity, and lifestyle modifications needed to combat the dual nutrition burden in school-age children. Over time, the MDM and the school health programs can be modified and they can develop the capacity to undertake screening for detection of under- and overnutrition; it will then be possible to provide focused care and monitor improvement in the nutritional status of school-age children.

## Conclusion

By providing BMI-for-age standards, the WHO has enabled an effective monitoring of growth trajectory during the critical 0–23 months of age in relation to infant and young child feeding, so that early interventions to correct wrong practices can be taken up. BMI-for-age standards help in the assessment of wasting and overnutrition in short-statured (0–18 years) children.

Recognizing the diversity of standards used, lacunae in availability, and reporting of data on overnutrition in countries, the SDG set targets as “halting the rise in overnutrition in children” without defining the standard to be used. This enables countries to use available data on the prevalence of overnutrition (irrespective of the standards used) to monitor ongoing efforts for prevention, early detection, and effective management of overnutrition.

If a uniform norm of BAZ > +1 was used for defining overnutrition, the prevalence of overnutrition in preschool children in India was high (almost similar to the prevalence of undernutrition). Currently, in India, nutrition programs for preschool children are focused on the management of undernutrition; it might be difficult to add on a component focused on combating overnutrition in large number of preschool children.

In the Indian context, it is preferable to define overnutrition as BAZ > +2 in the 0–18-year age group. If this were done, it will be possible to reorganize the ongoing ICDS, MDM, and school health programs over time, take-up integrated intervention aimed at early detection, and effective management of both the under- and overnutrition in 0–18-year children.

## Data Availability Statement

Publicly available datasets were analyzed in this study. This data can be found here: https://data.gov.in/search/site?query=annual+health+survey, http://www.iipsindia.org.

## Author Contributions

PR prepared the study design and tabulation plan. KK undertook the data cleaning and analysis and tabulation. All authors were involved in interpretation of the results and drafting the paper and approved the final version of the manuscript.

## Funding

This study was funded by our own institution Nutrition Foundation of India.

## Conflict of Interest

The authors declare that the research was conducted in the absence of any commercial or financial relationships that could be construed as a potential conflict of interest.

## Publisher's Note

All claims expressed in this article are solely those of the authors and do not necessarily represent those of their affiliated organizations, or those of the publisher, the editors and the reviewers. Any product that may be evaluated in this article, or claim that may be made by its manufacturer, is not guaranteed or endorsed by the publisher.
